# The importance of sizing in sutureless valves

**DOI:** 10.1093/icvts/ivac206

**Published:** 2022-08-04

**Authors:** Bart Meuris, Marie Lamberigts, Delphine Szecel

**Affiliations:** Department of Cardiac Surgery, University Hospitals Leuven, Leuven, Belgium; Department of Cardiac Surgery, University Hospitals Leuven, Leuven, Belgium; Department of Cardiac Surgery, University Hospitals Leuven, Leuven, Belgium

**Keywords:** Aortic valve, Bioprosthesis, Sutureless

This year, the Perceval sutureless valve is reaching its 15th anniversary. The first-in-man trial of this tissue valve was performed in 2007 and later completed with larger prospective trials for CE approval and initiation of commercial use [1, 2]. Since then, many centres across the world are using this tissue valve on a regular basis in a wide variety of patients with aortic valve disease. At this moment, Perceval is the only truly sutureless valve on the market, allowing aortic valve replacement without the use of a single suture that has to be knotted. Roughly estimated, around 75 000 valves have been implanted worldwide at this moment.

Around 2016–2017, ∼9 years after the first-in-man experience, the manufacturer decided to change their advice towards sizing of the prosthesis. After the observation of high pacemaker rates in the largest valve size (XL size) and some isolated cases of stent invagination due to oversizing, a new advice was given to use the commercial valve sizers differently. The actual valve is still slightly bigger in diameter compared to the white side of the corresponding sizer, so if this side fits into annulus with slight resistance, this is the correct size to choose. In the meantime, additional evidence exists that demonstrates the clear negative effects of oversizing this nitinol-based, sutureless valve [3].

Fabre *et al.* [4] are to be congratulated on their correct reporting of their overall experience and outcome with this technology. In the article, the authors focus specifically on the need for permanent pacemaker implantation, early after valve surgery using this sutureless valve. Two chronological study cohorts were defined: the experience before and after 2016. The new sizing strategy and which elements were changed are well described in the article. The need for pacemaker implantation decreased significantly from 16% to 5.9%.

We recently published our experience with Perceval, also looking at 2 cohorts in time, namely before and after 2017 [5]. A similar observation of a significantly decreased need for postoperative pacemakers from 11% to 6% was made, strengthening the observation made by Fabre. Regarding the analysis of the reasons why this pacemaker rate drops, we certainly agree with the new sizing method as an important factor in avoiding conduction disturbance after placement of the valve. We showed that just by downsizing by 1 size, the higher pacemaker rates disappear. The effect of the balloon dilation and the effect of the height of the Perceval positioning are more debatable in our opinion. Correct and complete decalcification on the other hand—as also mentioned by Fabre *et al.*—is important to obtain a good result with sutureless technology.

In conclusion, the observation made in this article is correct and corresponds to experiences in other centres. The main driver behind the decrease in pacemaker rate, however, in our opinion, is the new sizing method. The effect of ballooning and the higher implant position is open for discussion. The recommendation towards placing the guiding suture at a maximum of 2 mm below the annulus works fine for many users across the world. Correct and complete decalcification stays important obviously. Respecting all these steps and being familiar with the sizing, sutureless valves can offer many benefits to surgical patients, both in single aortic valve replacement (done minimally invasive) as in combined cases. This way, surgical aortic valve replacement offers a stable and safe result, with great haemodynamics in many patients: a result that is still highly competitive to the outcome seen in real-world transcatheter treatment.
